# Prediction models for sleep quality among frontline medical personnel during the COVID-19 pandemic: cross-sectional study based on internet new media

**DOI:** 10.3389/fpubh.2025.1406062

**Published:** 2025-03-26

**Authors:** Shangbin Huang, Qingquan Chen, Shengxun Qiu, Rongrong Dai, Ling Yao, Jiajing Zhuang, Zhijie Wu, Yifu Zeng, Jimin Fan, Yixiang Zhang

**Affiliations:** ^1^The Second Affiliated Hospital of Fujian Medical University, Quanzhou, China; ^2^The School of Public Health, Fujian Medical University, Fuzhou, China; ^3^The School of Clinical Medicine, Fujian Medical University, Fuzhou, China; ^4^Cyberspace Institute of Advanced Technology, Guangzhou University, Guangzhou, China

**Keywords:** sleep quality, medical personnel, COVID-19, machine learning sleep quality, deep learning

## Abstract

**Background:**

The factors associated with sleep quality among medical personnel providing support on the frontline during the height of the COVID-19 pandemic remain unclear, and appropriate predictive and screening tools are lacking. This study was designed and conducted to investigate whether factors such as weight change, job title, and tea consumption influence the sleep quality of these workers. Additionally, the study aims to develop predictive models to analyze the sleep problems experienced by healthcare workers during periods of epidemic instability, and to provide relevant data and tools to support effective intervention and prevention strategies.

**Methods:**

A cross-sectional study was conducted from June 25 to July 14, 2022, using a self-administered general information questionnaire and the Pittsburgh Sleep Quality Index (PSQI) to investigate the sleep quality of medical personnel providing aid in Shanghai. The relevant influencing factors were obtained via univariate analysis and multivariate stepwise logistic regression analysis, and 80% of the data were used in the training-test set (*n* = 1,060) and 20% were used in the independent validation set (*n* = 266). We used snowball sampling to establish the six models of logistics (LG), deep learning (DL), naïve Bayes (NB), artificial neural networks (ANN), random forest (RF), and gradient-boosted trees (GBT) and perform model testing.

**Results:**

Among the participants, 75.8% were female. Those under 35 years of age comprised 53.7% of the medical staff, while those over 35 years accounted for 46.3%. The educational background of the participants included 402 individuals with an associate degree (30.3%), 713 with a bachelor’s degree (53.8%), and 211 with a master’s degree or higher (15.9%).Weight, job title, and tea consumption during the aid period were the main factors influencing the sleep quality of medical personnel during the aid period. The areas under the curve (AUC) of LG, DL, NB, ANN, RF, and GBT were 0.645, 0.656, 0.626, 0.640, 0.551, and 0.582, respectively. The DL model has the best prediction performance (specificity = 86.1%, sensitivity = 45.5%) of all the models.

**Conclusion:**

During the height of the COVID-19 pandemic, the sleep quality of frontline medical personnel providing aid in Shanghai was influenced by multiple factors, and the DL model was found to have the strongest overall predictive efficacy for sleep quality.

## 1 Introduction

In February 2022, a COVID-19 outbreak occurred in Shanghai, and as of April 29, more than 50,000 local cases of novel coronavirus pneumonia were confirmed. From April 2022 to June 2022, frontline medical teams from Fujian Province assisted Shanghai in the fighting against the new coronavirus pneumonia, taking over several square-cabin hospitals in Shanghai to provide assistance in the treatment of the outbreak.

During the COVID-19 pandemic, the general population experienced increased psychological symptoms, including stress, depression, and anxiety, which negatively impacted sleep quality (1–3). Healthcare workers were particularly affected, with many reporting anxiety, depression, and insomnia. Studies showed that sleep problems were closely linked to these psychological issues, with healthcare workers experiencing higher rates of sleep disturbances and even PTSD compared to other professions (4–6). Increased exposure to COVID-19 risks contributed to greater psychological burdens for healthcare workers, leading to higher levels of self-reported anxiety and depression (7, 8). Poor sleep quality negatively affects both the physical and mental health of medical personnel, and it has been linked to increased safety risks (9, 10). For clinical staff, adequate sleep is crucial for maintaining optimal health, performance, and immune function (11). However, sleep and psychological issues among healthcare workers have not been adequately addressed, despite the severe impact of the pandemic on their well-being (12).

The pandemic overwhelmed healthcare systems, highlighting the need for targeted stress management and social support interventions for healthcare workers (13). Improving sleep quality through scientific and targeted management tools is essential to support healthcare workers’ physical and mental health, enabling them to better cope with the challenges of frontline work (31).

Machine learning (ML) and artificial intelligence (AI) have shown promise in healthcare, particularly in developing predictive models for sleep quality. These models have been successfully applied in areas such as sleep staging and sleep quality prediction during the COVID-19 pandemic (14–16). Machine learning, including predictive models based on short sleep questionnaires, has demonstrated potential for accurately identifying sleep disturbances, offering insights that could improve sleep health for healthcare workers (17).Thus, machine learning can be utilized to predict sleep problems, enhance our understanding of the sleep issues healthcare workers may face, and provide a solid foundation for effective solutions.

Previous studies have examined factors affecting the sleep quality of medical personnel during the COVID-19 pandemic. However, due to the varied working conditions, no studies have developed or validated sleep quality prediction models specifically for frontline medical personnel on assignment. Exploring the predictors of sleep quality affecting frontline medical personnel on assignment support and developing and validating sleep quality prediction models using machine learning methods such as LG, DL, NB, ANN, RF and GBT would be beneficial to help address the above issues.

This study was designed and conducted to investigate the sleep quality of frontline healthcare workers and the factors influencing it in the context of the fight against novel coronavirus pneumonia in Shanghai. Additionally, the study sought to develop a predictive model to better understand the sleep problems these healthcare workers may encounter during volatile public health outbreaks, providing relevant data and tools to support effective intervention and prevention strategies.

## 2 Methods

### 2.1 Study design

This cross-sectional study was conducted using a snowball sampling method to select 2024 administrators, nurses, clinicians, and other (medical technology, pharmacy, and testing) personnel who were part of the frontline medical workforce fighting novel coronavirus pneumonia in Shanghai from April 2022 to June 2022. The inclusion criteria included: 1. being administrative, nurses, clinicians, and other (medical, pharmacy, and laboratory) personnel who participated in front-line medical work against novel coronavirus pneumonia; 2. performing frontline clinical work for ≥1 week; 3. being located in various cities in Fujian Province; and 4. finishing the questionnaire within 10 min. The exclusion criteria included: 1. being non-working medical personnel; 2. not being from Fujian; 3. questionnaire filling time too short (<100 s) or too long (>10 min); and 4. questionnaire answers were obviously abnormal (age filled in 10 years old).We excluded a total of 698 respondents, and 1,326 respondents were ultimately included in the analysis.

### 2.2 Data collection

A questionnaire was dropped to the respondents via social media, including general information about the study participants and The Pittsburgh Sleep Quality Index (PSQI). The questionnaires were collected during the isolation period following the medical staff’s return from Shanghai to Fujian. The purpose of the survey and the requirements for completing the questionnaire were stated at the beginning of the questionnaire. After the questionnaires were collected, they were manually checked and invalid questionnaires were excluded.

### 2.3 Observation indicators

(1) The general information questionnaire included age, gender, address, marital status, education, title, type of work, length of service, and other basic information.
(2) Sleep status scale (last month): The Pittsburgh Sleep Quality Index (PSQI) was developed by Dr. Buysse, a psychiatrist at the University of Pittsburgh, in 1989 to assess overall sleep quality in seven areas: subjective sleep quality, sleep onset latency, sleep duration, sleep efficiency, sleep disturbances, use of sleep medications, daytime dysfunction, and global PSQI score. When the PSQI score is higher than 5, this indicates that there is a problem with sleep quality, and the higher the score, the worse the sleep quality. The higher the score, the worse the sleep quality. When the subject scored >1 on 7 aspects of the PSQI scale (subjective sleep quality, sleep onset latency, sleep duration, sleep efficiency, sleep disorder, hypnotic medication, and daytime dysfunction), it indicated a sleep problem.

### 2.4 Data analysis

The count data were expressed as frequencies and percentages, and the measurement data were expressed as means ± standard deviations (*x ± s*). A chi-square test (*χ^2^*) was used and the correlations of all variables were determined using Pearson correlation coefficients. The factors influencing sleep quality were analyzed using a one-way analysis of variance and multiple stepwise logistic regression. The OR values and 95% confidence intervals were calculated, and *p* values<0.05 were considered statistically significant (2-sided test). R (4.2.0) statistical software was used for data analysis.

### 2.5 Model building

The data were preprocessed for data normalization (including sample scale normalization, sample-by-sample mean subtraction, and normalization) and data whitening. Afterward, we performed data visualization and eigenvalue engineering on the data. Six models were built using LG, DL, NB, ANN, RF, and GBT. The six algorithms applied in this study are widely used supervised learning techniques capable of handling both numerical and categorical features. These models have a solid research foundation in the field of sleep problem prediction. Additionally, these models are well-known to users, which facilitates their adoption, application, and future customization upgrades. All models were trained using weight change, professional job titles, tea drinking during assistance to predict the sleep problems faced by these healthcare workers. The included data were randomly divided into a training-test set (80%, *n* = 1,060) and an independent validation set (20%, *n* = 266). To avoid overftting and improve the model, we used 10-fold cross-validation in the training-test set. In this process, all the data is divided into 10 parts and then each part is used as a validation set and the other parts are used as a training set for training and validation, which is repeated 10 times each time using a different validation set.

Samples from these six models were used in the training-test set as well as the independent validation set to test the model with the seven metrics of AUC, accuracy, sensitivity, specificity, precision, F1-score, and KAPPA. The area under the curve (AUC) values of the six models were then evaluated. The higher AUC closer to 1 indicates better performance.

### 2.6 Ethical review

This questionnaire was approved by the Medical Ethics Committee of the Second Affiliated Hospital of Fujian Medical University (IRB No. 2021–309), and all respondents were aware of and agreed to participate in this survey.

## 3 Results

### 3.1 Demographic characteristics

Participants were aged 23–58, mostly female, from Fuzhou, Xiamen, Zhangzhou, married, in medical/nursing roles, with undergraduate/college degrees, over 3 years of work (most >10 years), and mostly held various job titles: junior (50.2%), mid-level (32.0%) (Table 1).

**Table 1:** Demographic characteristics of the respondents in this survey (*N* = 1,326).

Characteristic	Contents	Participants, n (%)
Age	<35	712 (53.7)
	≥35	614 (46.3)
Gender	Male	321 (24.2)
	Female	1,005 (75.8)
Residence	Fuzhou	574 (43.3)
	Longyan	22 (1.7)
	Nanping	68 (5.1)
	Ningde	67 (5.1)
	Putian	88 (6.6)
	Quanzhou	72 (5.4)
	Sanming	70 (5.3)
	Xiamen	249 (18.8)
	Zhangzhou	116 (8.7)
Marital status	Unmarried	467 (35.2)
	Married	820 (61.8)
	Widowed	36 (2.7)
	Divorced	3 (0.2)
Education	Associate degree	402 (30.3)
	Bachelor’s degree	713 (53.8)
	Master’s degree and above	211 (15.9)
Professional job titles	None	39 (2.9)
	Junior	665 (50.2)
	Mid-level	424 (32.0)
	Deputy senior	166 (12.5)
	Senior	32 (2.4)
Type of work	Administrative personnel	34 (2.6)
	Nurse	911 (68.7)
	Doctor	289 (21.8)
	Medical technician	92(6.9)
Length of job (years)	0~2	113 (8.5)
	3~5	260 (19.6)
	6~10	347 (26.2)
	>10	606 (45.7)

### 3.2 Some characteristics of the situation before participating in the aid process

The chi-square test showed that some of the medical personnel’s previous condition characteristics such as BMI, dietary pattern, exercise routine, alcohol consumption, sun exposure, tea drinking, smoking, and underlying diseases were not statistically significant in association with sleep quality before participating in the aid process (Table 2).

**Table 2:** Some characteristics of the situation before participating in the rescue.

Variables	*N* = 1,326	Sleep quality		Univariate analysis		
		Good (PSQI scores≤5)	Poor (PSQI scores>5)	χ^2^	Degrees of freedom	*p*
BMI before assistance						
18.5 kg/m^2^	145	14(13.0)	131(10.8)	0.537	3	0.911
18.5~23.9 kg/m^2^	878	70(64.8)	808(66.3)			
24.0~27.9 kg/m^2^	261	21(19.4)	240(19.7)			
≥28.0 kg/m^2^	42	3(2.8)	39(3.2)			
Meal regularity before assistance						
No	953	85(78.7)	868(71.3)	2.360	1	0.124
Yes	373	23(21.3)	350(28.7)			
Daily exercise duration						
<30 min/day	611	41(38.0)	570(46.8)	3.235	2	0.198
30~60 min/day	545	50(46.3)	495(40.6)			
>60 min/day	170	17(15.7)	153(12.6)			
Drinking before assistance						
No	909	73(67.6)	836(68.6)	4.601	3	0.203
<15 g/day	352	29(26.9)	323(26.5)			
15~25 g/day	45	2(1.9)	43(3.5)			
>25 g/day	20	4(3.7)	16(1.3)			
Sun exposure before assistance						
No	715	66(61.1)	649(53.3)	3.352	2	0.187
<30 min/day	380	23(21.3)	357(29.3)			
≥30 min/day	231	19(17.6)	212(17.4)			
Tea drinking before assistance						
No	514	50(46.3)	464(38.1)	2.861	3	0.414
Occasionally (1~2/week)	535	38(35.2)	497(40.8)			
Frequently (3~5/week)	200	14(13.0)	186(15.3)			
Every day	77	6(5.6)	71(5.8)			
Smoking before assistance						
No	1,205	105(97.2)	1,100(90.3)	5.904	3	0.116
<5 cigarettes/day	85	2(1.9)	83(6.8)			
5~10 cigarettes/day	24	1(0.9)	23(1.9)			
>10 cigarettes/day	12	0(0.0)	12(1.0)			
Basic diseases						
No	1,175	102(94.4)	1,073(88.1)	8.140	7	0.320
Diabetes	15	0(0.0)	15(1.2)			
Chronic kidney disease	6	0(0.0)	6(0.5)			
Coronary heart disease	2	0(0.0)	2(0.2)			
Hyperlipidemia	21	1(0.9)	20(1.6)			
Hypertension	36	2(1.9)	34(2.8)			
Malignant tumors	3	1(0.9)	2(0.2)			
Other	68	2(1.9)	66(5.4)			

### 3.3 Comparison of scores on seven components of the PSQI among medical personnel with different working ages

There was no statistical significance in scores between the different working age groups except in terms of sleep efficiency and the use of sleep medications (*p* = 0.054 and 0.384, respectively). The 0–2 years working age group had the best sleep quality (1.35 ± 0.92), sleep onset latency (1.57 ± 1.17), sleep duration (1.35 ± 0.94), and sleep disturbances (1.30 ± 0.93) and the lowest Global PSQI score (9.64 ± 4.67), which indicates that those with 0–2 years of service had the best subjective sleep quality, the shortest time to fall asleep, the longest sleep duration, and the least sleep disturbances. Those with >10 years of service had the worst subjective sleep quality and the shortest sleep duration. The final PSQI total score shows that the overall quality of sleep was better for those with 0–2 years of service (9.64 ± 4.67), while the Global PSQI score was worse for those with 6–10 years of service (11.72 ± 3.87), reflected in the lowest scores for time to fall asleep, sleep disturbances, and daytime dysfunction (Table 3).

**Table 3:** Comparison of scores on seven components of the PSQI among medical personnel with different lengths of job in this survey ( $\bar{x}$ ±S).

	Subjective sleep quality	Sleep onset latency	Sleep duration	Sleep efficiency	Sleep disturbances	Use of sleep medications	Daytime dysfunction	Global PSQI score
0~2	1.35 ± 0.92	1.57 ± 1.17	1.35 ± 0.94	1.89 ± 1.11	1.30 ± 0.93	0.71 ± 1.00	1.48 ± 1.10	9.64 ± 4.67
3~5	1.61 ± 0.88	1.95 ± 0.98	1.65 ± 0.89	1.74 ± 1.17	1.53 ± 0.82	0.91 ± 1.15	1.73 ± 1.02	11.12 ± 4.13
6~10	1.72 ± 0.85	2.08 ± 0.93	1.68 ± 0.88	1.79 ± 1.11	1.66 ± 0.78	0.98 ± 1.11	1.82 ± 0.93	11.72 ± 3.87
>10	1.79 ± 0.89	2.01 ± 0.98	1.83 ± 0.88	1.67 ± 1.07	1.59 ± 0.81	0.89 ± 1.10	1.78 ± 0.99	11.56 ± 3.92
The global score	1.70 ± 0.89	1.98 ± 0.99	1.71 ± 0.90	1.73 ± 1.11	1.57 ± 0.82	0.90 ± 1.10	1.76 ± 0.99	11.35 ± 4.05
*F*	25.100	11.220	27.670	3.730	8.153	0.758	5.917	15.67
*P*	<0.001	<0.001	<0.001	0.054	0.004	0.384	0.015	<0.001

Compared with 0~2.

### 3.4 Comparison of scores on seven components of the PSQI among medical personnel with different education levels

There was no statistically significant relationship with sleep quality among people with different education levels in terms of sleep duration (*p* = 0.746), use of sleep medications (*p* = 0.242), and Global PSQI score (*p* = 0.286). Those with college degrees had the lowest scores in the three aspects of sleep efficiency (1.59 ± 1.08), sleep disturbances (1.54 ± 0.75), and daytime dysfunction (1.66 ± 0.91) while having the highest scores in the two aspects of subjective sleep quality (1.85 ± 0.83) and sleep onset latency (2.13 ± 0.91), implying that those with college degrees had the highest sleep efficiency, encountered the least sleep disturbances, and had the least daytime dysfunction but had the worst subjective sleep quality and the longest sleep time; those with a bachelor’s degree had the best subjective sleep quality (1.60 ± 0.88); those with master’s degree and above had the shortest sleep time (1.87 ± 1.07) but the lowest sleep efficiency (1.82 ± 1.12) and also encountered sleep disturbances (1.70 ± 0.91) and daytime dysfunction (2.02 ± 1.03) the most frequently (Table 4).

**Table 4:** Comparison of scores on seven components of the PSQI among medical personnel with different educational backgrounds in this survey ( $\bar{x}$ ±S).

	Subjective sleep quality	Sleep onset latency	Sleep duration	Sleep efficiency	Sleep disturbances	Use of sleep medications	Daytime dysfunction	Global PSQI score
Associate degree	1.85 ± 0.83	2.13 ± 0.91	1.77 ± 0.87	1.59 ± 1.08	1.54 ± 0.75	0.95 ± 1.12	1.66 ± 0.91	11.48 ± 3.73
Bachelor’s degree	1.60 ± 0.88	1.93 ± 1.00	1.65 ± 0.90	1.79 ± 1.11	1.55 ± 0.82	0.80 ± 1.04	1.73 ± 1.01	11.06 ± 4.03
Master’s degree and above	1.74 ± 0.96	1.87 ± 1.07	1.80 ± 0.91	1.82 ± 1.12	1.70 ± 0.91	1.14 ± 1.24	2.02 ± 1.03	12.10 ± 4.59
The global score	1.70 ± 0.89	1.98 ± 0.99	1.71 ± 0.90	1.73 ± 1.11	1.57 ± 0.82	0.90 ± 1.10	1.76 ± 0.99	11.35 ± 4.05
*F*	5.558	12.120	105.000	8.597	4.361	1.371	15.61	1.138
*P*	0.019	<0.001	0.746	0.003	0.037	0.242	<0.001	0.286

Compared with associate degree.

### 3.5 Comparison of the detection rate of seven sleep problems in working hours over 8 h per day

The detection rates of all seven sleep problems were statistically different between those who worked more than 8 h per day compared to those who worked less than 8 h per day. Among the seven sleep problems, the greatest difference in the detection rate of sleep onset disorder [499 (72.5%)] was encountered in the population working more than 8 h per day compared to those working less than 8 h per day, followed by daytime dysfunction [462 (67.2%)] and subjective sleep quality [458 (66.6%)]. Only in the case of hypnotic drug use was the score of the population working less than 8 h per day higher than that of the population working more than 8 h. The comparison of the detection rates of the seven sleep problems in the case of working more than 8 h per day is shown in Table 5.

**Table 5:** Comparison of the detection rate of 7 sleep problems for those working over 8 h daily in this survey [n (%)].

Variables	Subjective sleep quality	Sleep onset latency	Sleep duration	Sleep efficiency	Sleep disturbances	Use of sleep medications	Daytime dysfunction
Working hours ≤8	230(33.4)	189(27.5)	282(41.0)	299(43.5)	306(44.5)	415(60.3)	226(32.8)
Working hours >8	458(66.6)	499(72.5)	406(59.0)	389(56.5)	382(55.5)	273(39.7)	462(67.2)
df	1	1	1	1	1	1	1
χ^2^	17.383	6.217	15.845	7.535	27.926	49.038	22.341
P	<0.001	0.013	<0.001	0.006	<0.001	<0.001	<0.001

Each PSQI component score > 1 point indicates a sleep problem.

### 3.6 Univariate analysis of factors affecting sleep quality

The univariate analysis showed that gender, education, weight change, type of work, job title, length of service, substance use during assistance, position support, and tea drinking during assistance were statistically associated with sleep quality (all *p* < 0.05). Sleep quality was poorer among those who were female, had a university degree, lost weight, were doctors, had a junior title, had more than 10 years of work experience, and drank tea during assistance. However, the use of drugs during assistance had little effect on the quality of sleep. The sleep quality was generally poorer among those in any support positions (Table 6).

**Table 6:** Univariate analysis of factors affecting sleep quality [number] among medical personnel in this survey.

Variables	*N* = 1,326	Sleep Quality		Univariate analysis		
		Good (PSQI scores≤5)	Poor (PSQI scores>5)	χ^2^	Degrees of freedom	*p*
Age						
<35	712	66(61.1)	646(53.0)	2.286	1	0.131
≥35	614	42(38.9)	572(47.0)			
Gender						
Male	321	40(37.0)	281(23.1)	9.799	1	0.002
Female	1,005	68(63.0)	937(76.9)			
Education						
Associate degree	402	21(19.4)	381(31.3)	6.684	2	0.035
Bachelor’s degree	713	66(61.1)	647(53.1)			
Master’s degree and above	211	21(19.4)	190(15.6)			
BMI during assistance						
18.5 kg/m^2^	252	15(13.9)	237(19.5)	2.781	3	0.427
18.5 ~ 23.9 kg/m^2^	874	76(70.4)	798(65.5)			
24.0 ~ 27.9 kg/m^2^	175	16(14.8)	159(13.1)			
≥28.0 kg/m^2^	25	1(0.9)	24(2.0)			
Weight change						
Lighten	1,056	64(59.3)	992(81.4)	32.372	2	<0.001
No	203	36(33.3)	167(13.7)			
Heavier	67	8(7.4)	59(4.8)			
Type of work						
Administrative personnel	34	0(0.0)	34(2.8)	45.290	3	<0.001
Nurse	92	23(21.3)	69(5.7)			
Doctor	911	55(50.9)	856(70.3)			
Medical technician	289	30(27.8)	259(21.3)			
Professional job titles						
None	39	11(10.2)	28(2.3)	23.802	4	<0.001
Junior	665	55(50.9)	610(50.1)			
Mid-level	424	32(29.6)	392(32.2)			
Deputy senior	166	8(7.4)	158(13.0)			
Senior	32	2(1.9)	30(2.5)			
Length of job (years)						
0~2	113	27(25.0)	86(7.1)	44.060	3	<0.001
3~5	260	23(21.3)	237(19.5)			
6~10	347	17(15.7)	330(27.1)			
>10	606	41(38.0)	565(46.4)			
Marital status						
Unmarried	467	50(46.3)	417(34.2)	6.629	3	0.085
Married	820	55(50.9)	765(62.8)			
Widowed	36	3(2.8)	33(2.7)			
Divorced	3	0(0.0)	3(0.2)			
Medical assistance history						
No	638	66(61.1)	572(47.0)	7.399	1	0.007
Yes	688	42(38.9)	646(53.0)			
Support positions						
Designated hospital general ward	348	24(22.2)	324(26.6)	14.965	4	0.005
Fever clinics	343	43(39.8)	300(24.6)			
Designated hospital ICU	32	3(2.8)	29(2.4)			
Nucleic acid sampling and detection	308	14(13.0)	294(24.1)			
Other	295	24(22.2)	271(22.2)			
Work shift						
Day shift	448	40(37.0)	408(33.5)	0.674	2	0.714
Night shift	32	3(2.8)	29(2.4)			
Change shifts	846	65(60.2)	781(64.1)			
Daily working hours in contaminated areas						
≤8	1,258	105(97.2)	1,153(94.7)	0.861	1	0.353
>8	68	3(2.8)	65(5.3)			
Days off per week						
≤2	1,283	104(96.3)	1,179(96.8)	<0.001	1	1.000
>2	43	4(3.7)	39(3.2)			
Tea drinking during assistance						
No	612	66(61.1)	546(44.8)	10.654	3	0.014
Occasionally (1-2/week)	431	25(23.1)	406(33.3)			
Frequently (3-5/week)	191	12(11.1)	179(14.7)			
Every day	92	5(4.6)	87(7.1)			
Smoking during assistance						
No	1,210	103(95.4)	1,107(90.9)	4.977	3	0.174
<5 cigarettes/day	73	2(1.9)	71(5.8)			
5~10 cigarettes/day	26	3(2.8)	23(1.9)			
>10 cigarettes/day	17	0(0.0)	17(1.4)			
Basic diseases						
No	1,175	102(94.4)	1,073(88.1)	3.359	1	0.067
Yes	151	6(5.6)	145(11.9)			
Frequency of contacting COVID-19 patients						
Every day	186	15(13.9)	171(14.0)	3.495	2	0.174
Every few days	277	30(27.8)	247(20.3)			
No contact	863	63(58.3)	800(65.7)			

### 3.7 Multi-factor analysis of factors influencing sleep quality in medical personnel

**Figure 1:**
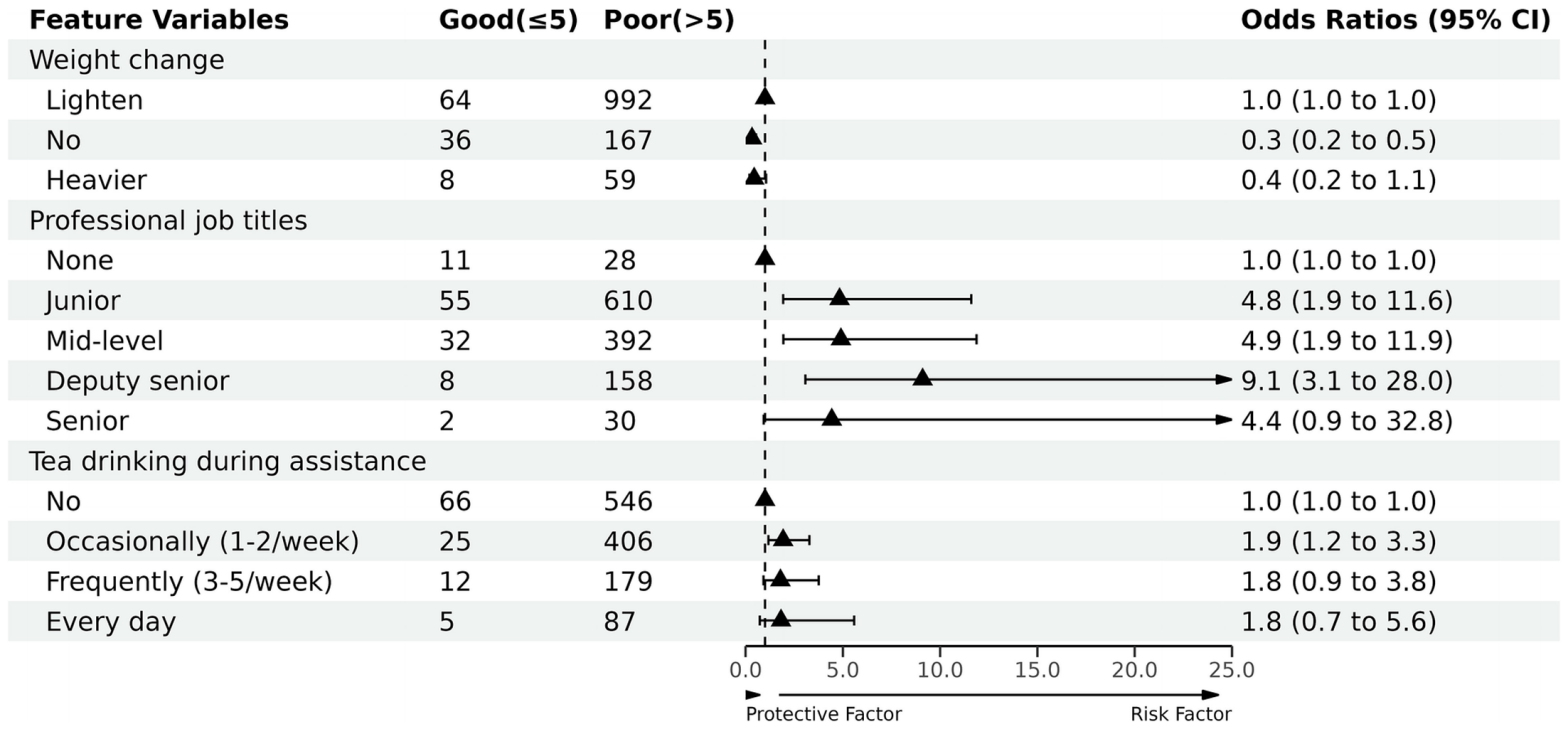
Forest plot of odds ratios (ORs) for 3 predictors included in the prediction mode.

The factors with *p* < 0.10 in the univariate analysis, gender, education, weight change, job type, job title, length of service, marital status, drug use during assistance, position support, tea drinking during assistance, and underlying disease status, were included in the multiple stepwise logistic regression analysis. The seven factors of gender, weight change, job type, job title, support position, tea consumption during assistance, and underlying disease were further analyzed according to AIC, and the results show that weight change, job title, and tea consumption during assistance are the main predictors of sleep quality among medical personnel during assistance, with statistically significant differences (*p* < 0.05) (Table 7). Further regression analysis of these three showed that the factors of no change in weight or weight gain were favorable to sleep quality, while having junior, intermediate, associate, and senior titles and drinking tea during assistance were all risk factors for sleep quality, with deputy senior (OR 9.1, 95% CI 3.1–28.0) in particular having the greatest impact on sleep quality (Figure 1).

**Table 7:** Results of the multivariate stepwise logistic regression analysis of factors related to sleep quality among medical personnel in this survey.

Predictor	OR	95% CI	*p*
Gender			
Male	1.00		
Female	1.78	0.99–3.19	0.054
Weight change			
Lighten	1.00		
No	0.33	0.20–0.54	<0.001
Heavier	0.44	0.20–1.05	0.045
Type of work			
Administrative personnel	1.00		
Nurse	0.00	0.00–0.00	0.979
Doctor	0.00	0.00–6681782.21	0.981
Medical technician	0.00	0.00–0.00	0.980
Professional job titles			
None	1.00		
Junior	4.83	1.93–11.60	<0.001
Mid-level	4.90	1.94–11.87	<0.001
Deputy senior	9.10	3.07–28.04	<0.001
Senior	4.43	0.93–32.77	0.087
Support positions			
Designated hospital general ward	1.00		
Fever clinics	0.60	0.33–1.07	0.087
Designated hospital ICU	0.90	0.25–4.64	0.889
Nucleic acid sampling and detection	2.09	1.01–4.52	0.051
Other	0.79	0.42–1.48	0.462
Tea drinking during assistance			
No	1.00		
Occasionally (1–2/week)	1.93	1.17–3.28	0.012
Frequently (3–5/week)	1.79	0.92–3.76	0.101
Every day	1.82	0.73–5.58	0.237
Basic diseases			
No	1.00		
Yes	2.33	1.01–6.40	0.069

### 3.8 Performance of prediction model

**Figure 2:**
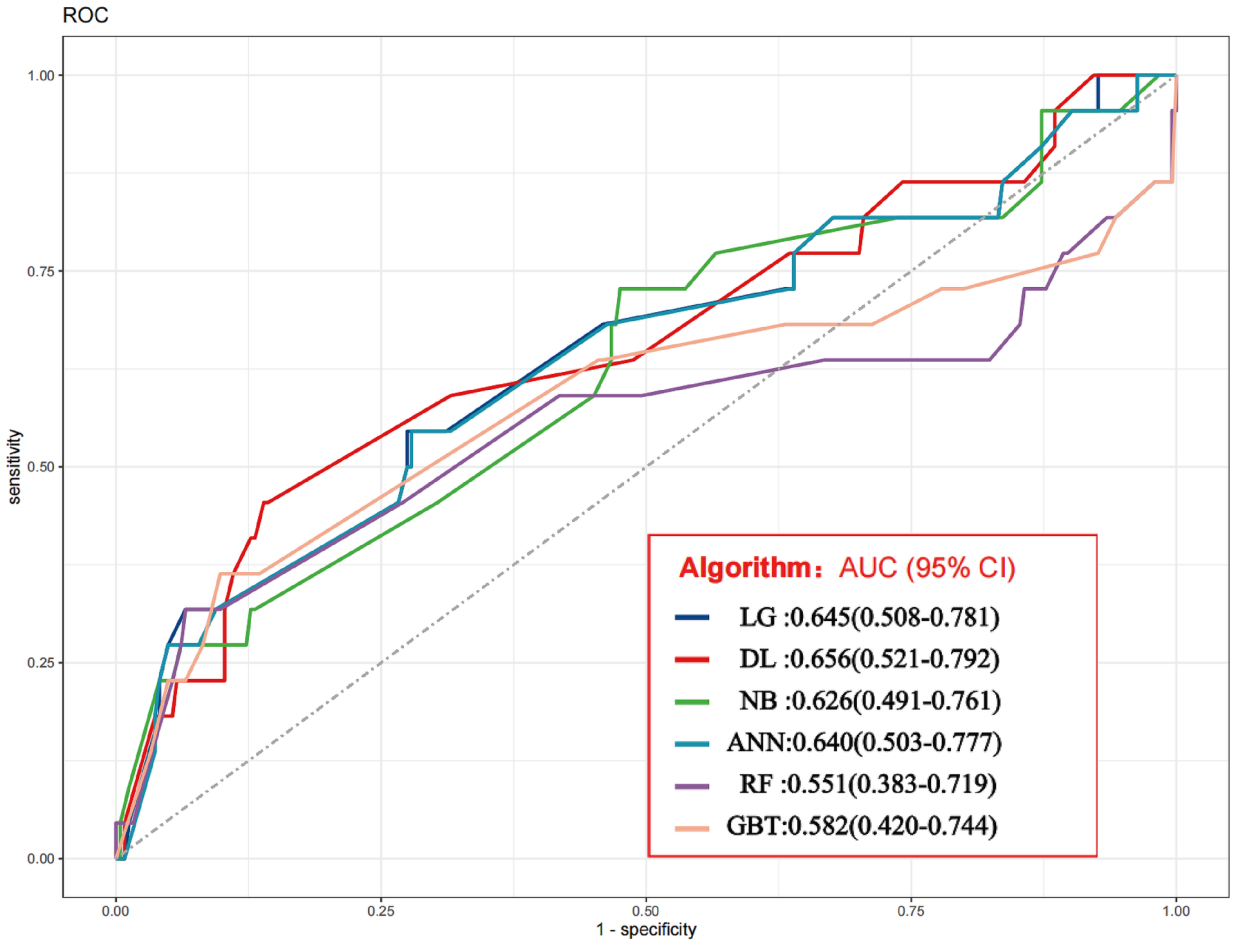
The ROC curves of the six model.

Following sufficient training, the six models were utilized on the validation set. The hyperparameters for the all six models are provided in Supplementary Table 1. The seven metrics of AUROC, accuracy, sensitivity, specificity, precision, F1-score, and KAPPA of the tested models were used to perform the testing of the six models LG, DL, NB, ANN, RF, and GBT. The ROC curves of the six models are analytically evaluated as follows (Figure 2), where LG: AUC = 0.645 (0.508–0.781), DL: AUC = 0.656 (0.521–0.792), NB: AUC = 0.626 (0.491–0.761), ANN: AUC = 0.640 (0.503–0.777), RF: AUC = 0.551 (0.383–0.719), and GBT: AUC = 0.582 (0.420–0.744). It can be seen that the DL model has the largest AUC, so the DL model performed best with the following specific indicators: AUROC (95% CI): 0.656 (0.521–0.792), accuracy (95% CI): 0.827 (0.828–0.826), sensitivity (95% CI): 0.455 (0.663–0.246), specificity (95% CI): 0.861 (0.904–0.817), precision (95% CI): 0.227 (0.351–0.103), F1-score (95% CI): 0.303 (0.459–0.145), and KAPPA (95% CI): 0.217 (0.368–0.065) (Table 8). The DL model has the highest AUC value (0.656), but its low sensitivity (45.5%) limits its application in screening and makes it more suitable for confirming positive cases, especially in scenarios that require high specificity (Table 8).

**Table 8:** Performance metrics and practical application significance of six models.

Algorithm	Discrimination tests							Practical application significance
	AUROC (95% CI)	Accuracy (95% CI)	Sensitivity (95% CI)	Specificity (95% CI)	Precision (95% CI)	F1-score (95% CI)	KAPPA (95% CI)	
Logistic regression	0.645 (0.508–0.781)	0.711 (0.712–0.709)	0.545 (0.754–0.337)	0.725 (0.781–0.669)	0.152 (0.231–0.073)	0.238 (0.354–0.120)	0.124 (0.230–0.018)	Screening^a^
Deep learning	0.656 (0.521–0.792)	0.827 (0.828–0.826)	0.455 (0.663–0.246)	0.861 (0.904–0.817)	0.227 (0.351–0.103)	0.303 (0.459–0.145)	0.217 (0.368–0.065)	Confirming positive cases^b^
Naïve Bayes	0.626 (0.491–0.761)	0.541 (0.543–0.540)	0.727 (0.913–0.541)	0.525 (0.587–0.462)	0.121 (0.177–0.066)	0.207 (0.297–0.118)	0.077 (0.144–0.010)	Screening^c^
Artificial neural network	0.640 (0.503–0.777)	0.707 (0.708–0.705)	0.545 (0.754–0.337)	0.721 (0.778–0.665)	0.150 (0.228–0.072)	0.235 (0.350–0.119)	0.121 (0.226–0.016)	Screening^a^
Random forest	0.551 (0.383–0.719)	0.883 (0.884–0.883)	0.318 (0.513–0.124)	0.934 (0.965–0.903)	0.304 (0.492–0.116)	0.311 (0.502–0.120)	0.247 (0.433–0.062)	Confirming positive cases^b^
Gradient boosted trees	0.582 (0.420–0.744)	0.857 (0.858–0.856)	0.364 (0.565–0.163)	0.902 (0.939–0.864)	0.250 (0.400–0.100)	0.296 (0.468–0.124)	0.220 (0.388–0.051)	Confirming positive cases^d^

a Both specificity and sensitivity are at a moderate level.

b Not suitable for screening, but suitable for confirming positive cases, especially in scenarios that require high specificity.

c Suitable for preliminary screening, but further optimization is needed to reduce false positive results.

d Suitable for confirming positive cases, but further optimization is needed to improve sensitivity.

## 4 Discussion

### 4.1 Main results

Our results show that the sleep quality of medical personnel providing aid in Shanghai during the epidemic was influenced by factors such as weight change, job title, and tea consumption during the aid period. After model building, training, and validation, the DL model has the best performance for predicting the sleep quality of frontline medical personnel in outstation support. This model may assist in identifying individuals at risk of poor sleep quality. This study reveals that healthcare workers with fewer working years exhibit better sleep quality than their more experienced counterparts (*p* < 0.05), likely attributable to cumulative work-related stress and age-related physiological decline. Assessments of interpersonal and psychological stress responses showed higher scores among senior staff, indicating intensified interpersonal challenges and reduced stress tolerance with prolonged tenure. Notably, all participants displayed elevated PSQI scores (>11), underscoring the pervasiveness of sleep disturbances in this population. Additionally, individuals with undergraduate degrees had the best sleep quality, possibly because they worked in less stressful environments and had more psychological relief than those with postgraduate degrees.

In this study, the sleep quality of medical staff working more than 8 h a day was significantly lower than that of those working 8 h or less (*p* < 0.05), indicating that extended working hours negatively impact sleep quality. The data suggest that continuous overload can lead to burnout (18), which may create a bidirectional relationship (19) through psychological changes such as anxiety and depression. It is noteworthy that during the medical team’s deployment in Shanghai, task intensity increased sharply, and the average daily working hours were significantly longer than those typically encountered in their regular duties. This aligns with the findings of a cross-sectional study involving 1,510 male white-collar workers, which confirmed a dose–response relationship between working hours and the risk of sleep disorders (20). Existing evidence indicates that this vicious cycle ultimately compromises mental health and consistently reduces sleep quality (21, 22).

### 4.2 Analysis of influencing factors

Multiple stepwise logistic regression analysis revealed that weight change, job title, and tea consumption during assistance affected sleep quality. While previous studies suggest a link between obesity, poor sleep, and depression (23, 24), our findings show that weight gain may improve sleep quality in healthcare workers. This discrepancy could be due to our study’s small sample size. As they gain higher job titles, healthcare workers are often required to take on more important and urgent medical tasks at work. At the same time, due to the specialized and complex nature of medical knowledge, health care workers need to continuously learn and gain experience, and health care workers with lower job titles sometimes need to rely on the training and guidance of their supervisors to perform their work independently (25). Therefore, medical and nursing staff with higher titles need to learn and accumulate experience. As a result, health care workers with higher titles may experience higher work stress and be in a more stressful and anxious mental state when facing high-risk medical tasks for long periods of time, which may affect their sleep quality. Moreover, the prolonged nature of these high-stress responsibilities—such as extended shifts, complex medical tasks, and the emotional toll of dealing with critically ill patients—can lead to burnout, a well-established factor contributing to poor sleep quality. The heightened stress levels experienced by senior medical personnel may also result in difficulties “switching off” from work, making it harder for them to relax and fall asleep after intense working hours. As a result, they may experience disturbed sleep patterns, including difficulty falling asleep, frequent awakenings, and reduced sleep quality overall.

Tea contains caffeine, and drinking tea before bed can disrupt sleep and reduce sleep quality (26, 27). In fact, caffeine administration has been used as a model for insomnia (28). Medical personnel may be under prolonged stress, and when placed in an unfamiliar environment may be more in need of tea, coffee, and other refreshments. As Fujian is a region with a strong tea culture, the personnel providing aid in Shanghai were more inclined to choose to refresh with tea, thus leading to poor sleep quality; this degree of sleep deprivation, if experienced for longer than one night, may adversely affect daytime functioning (29). Poor sleep could be both a cause and a consequence of caffeinated beverage consumption. The affected medical personnel thus drink more tea, and so repeatedly enter the cycle of poor sleep quality. Secondly, regarding smoking and alcohol consumption, it is important to note that these behaviors might have been less prevalent or less impactful during the assistance period. For instance, smoking might not have been as common among the medical personnel participating in the study, or its effects on sleep quality might have been overshadowed by the more immediate stressors of the work environment. Additionally, alcohol consumption was likely restricted or prohibited during the assistance period due to the need for clear-headedness and professionalism in a high-stakes medical setting. As a result, the potential impact of alcohol on sleep quality was minimal. In conclusion, the significant impact of tea consumption on sleep quality during the assistance period can be attributed to its widespread use as a coping mechanism in a high-stress environment, while other lifestyle factors such as smoking and alcohol consumption did not significantly affect sleep quality.

### 4.3 Advantages

Previous articles have also used deep learning methods to predict sleep disturbances in asthma patients (30), but this is the first instance of the development and establishment of a prediction model for sleep quality in frontline healthcare workers involved in medical assistance. The prediction model we developed demonstrates outstanding specificity (true negative rate) but has low sensitivity (true positive rate). This indicates that the likelihood of misdiagnosis is lower than that of underdiagnosis when determining the presence of sleep disturbances. Given that the prognosis for sleep disturbances is generally better than for severe conditions like malignant tumors, the psychological stress caused by misdiagnosis can be considerable. Therefore, the risks associated with misdiagnosis are more harmful in this context. The application of a deep learning model (DL model) can help mitigate these risks. Consequently, when the model indicates a positive result, the presence of sleep disturbances should be highly suspected. This advantage helps in selecting individuals with poor sleep quality and intervening actively to reduce the adverse effects of reduced sleep quality in health workers. Therefore, the generated model is beneficial for understanding the sleep status of frontline medical workers providing assistance in Shanghai aid and also aids in the screening of sleep disturbances.

Our results indicate that by collecting only a few key variables, we can potentially predict the sleep quality of frontline personnel using deep learning (DL) technology. This predictive capability would be beneficial for monitoring the sleep quality of medical staff during future unknown pandemic situations or similar high-intensity work. Ultimately, it provides an effective tool to enhance both the sleep quality and overall quality of life for healthcare workers. Improved sleep can help these individuals maintain better psychological and physical well-being, enabling them to effectively manage frontline prevention efforts and sustain a healthy healthcare environment.

### 4.4 Limitations

There are some limitations to this study. Data collection dimensions: First, we used a snowball sampling method based on an online questionnaire, which makes our sample dependent on the online web environment and potentially prone to selection bias. This bias may cause the distribution of healthcare workers in the sample to be misaligned with the true demographic and occupational characteristics of the overall population, particularly concerning factors such as gender, age, and professional experience. Additionally, in this sample, all data were self-reported; as a result, we have no objective measures of sleep quality and participants’ insomnia and health status prior to HAI are unknown, which limits our interpretation of the results. Second, ours is a cross-sectional survey, which limits the ability to interpret the causal relationships between the different variables in this study and to determine associations and causality more precisely. In addition, due to the inevitable defects of the questionnaire via social media, some selection bias may be caused, which we hope to be further improved in future studies. Finally, because our study was limited to frontline medical personnel in Fujian Province who provided assistance in Shanghai.

## 5 Conclusion

This study shows that the sleep quality of frontline medical personnel in Fujian Province was affected by providing aid in Shanghai. Weight change, job title, and tea consumption during assistance were the main influencing factors. The DL model showed strong predictive power for sleep quality among frontline healthcare workers, but its low sensitivity limits its ability to accurately identify all sleep disturbances. While promising, the model should be used with caution and may require further validation and integration with other methods for improved accuracy.

Our findings may provide an effective tool for improving the sleep quality and overall quality of life of frontline healthcare workers during volatile public health epidemics. Our study will also help improve the sleep quality of healthcare workers in possible unexpected situations with long workloads and provides relevant suggestions to reduce the degree to which sleep quality is affected, improving the sleep quality of healthcare workers.

## Data Availability

The original contributions presented in the study are included in the article/Supplementary material, further inquiries can be directed to the corresponding authors.
