# Supplementary material for: Prediction models for sleep quality among frontline medical personnel during the COVID-19 pandemic: cross-sectional study based on internet new media
Source: Front Public Health. 2025 Mar 26;13:1406062. doi: 10.3389/fpubh.2025.1406062 (PMC11978626; doi:10.3389/fpubh.2025.1406062)
Supplement: Supplementary file 1 [file Table_1.DOCX]

**Supplementary Table 1**

Optimum parameter combination using grid search.

| **Model** | **Hyperparameters** | **Optimal value** |
| --- | --- | --- |
| Logistic regression | C | 1 |
|  | Penalty | L_2_ |
| Deep learning | Activation | Rectifier |
|  | Hidden Layer Sizes | 50 |
|  | Learning Rate | 0.005 |
|  | Adaptive Rate | TRUE |
|  | Momentum Start | 0 |
|  | Momentum Ramp | 1000000 |
|  | Momentum Stable | 0 |
|  | Nesterov Accelerated Gradient | TRUE |
|  | L1 | 1.0E-05 |
|  | L2 | 0 |
|  | Max W2 | 10 |
|  | Epochs | 10 |
|  | Compute Variable Importances | FALSE |
|  | Learning Rate Annealing | 1.0E-06 |
|  | Learning Rate Decay | 1 |
|  | Standardize | TRUE |
|  | Loss Function | Automatic |
|  | Distribution Function | AUTO |
|  | Stopping Rounds | 1 |
|  | Stopping Metric | AUTO |
|  | Stopping Tolerance | 0.001 |
|  | Missing Values Handling | MeanImputation |
|  | Max Runtime Seconds | 0 |
| Naïve Bayes | Alpha | 1 |
|  | Bandwidth | 0.1 |
|  | Minimum bandwidth | 0.1 |
|  | Number of kernels | 10 |
|  | Application grid size | 200 |
| Artificial neural network | Training cycles | 195 |
|  | Learning rate | 0.01 |
|  | Momentum | 0.9 |
|  | Maximum number of threads | 4 |
|  | Error epsilon | 1.0E-4 |
| Random forest | Max_depth | 10 |
|  | Minimal gain | 0.01 |
|  | Minimal leaf size | 2 |
|  | Minimal size for split | 4 |
|  | Number of prepruning alternatives | 3 |
|  | Prepruning alternatives | 3 |
|  | Subset ratio | 0.2 |
| Gradient Boosted trees | Number of trees | 50 |
|  | Maximum number of threads | 4 |
|  | Maximal depth | 5 |
|  | Min rows | 10 |
|  | Min split improvement | 1.0E-5 |
|  | Number of bins | 20 |
|  | Learning rate | 0.01 |
|  | Sample rate | 1.0 |
|  | Stopping rounds | 1 |
|  | Stopping tolerance | 0.001 |

*Note:* All options were left as default other than hyper-parameters in table.
